# New perspectives on the induction and acceleration of immune-associated thrombosis by PF4 and VWF

**DOI:** 10.3389/fimmu.2023.1098665

**Published:** 2023-02-28

**Authors:** Zhi-Yan Liu, Min-Xue Sun, Man-Qi Hua, Han-Xu Zhang, Guang-Yan Mu, Shuang Zhou, Zhe Wang, Qian Xiang, Yi-Min Cui

**Affiliations:** ^1^ Department of Pharmacy, Peking University First Hospital, Beijing, China; ^2^ Department of Pharmacy Administration and Clinical Pharmacy, School of Pharmaceutical Sciences, Peking University, Beijing, China; ^3^ Institute of Clinical Pharmacology, Peking University First Hospital, Beijing, China; ^4^ School of Basic Medicine and Clinical Pharmacy, China Pharmaceutical University, Nanjing, China

**Keywords:** heparin-induced thrombocytopenia, immune associated thrombosis, mechanism, platelet factor 4, Von Willebrand factor

## Abstract

Platelet factor 4 (PF4), also known as chemokine (C-X-C motif) ligand 4 (CXCL4), is a specific protein synthesized from platelet α particles. The combination of PF4 and heparin to form antigenic complexes is an important mechanism in the pathogenesis of heparin-induced thrombocytopenia (HIT), but vaccine-induced immune thrombotic thrombocytopenia (VITT) related to the COVID-19 vaccine makes PF4 a research hotspot again. Similar to HIT, vaccines, bacteria, and other non-heparin exposure, PF4 can interact with negatively charged polyanions to form immune complexes and participate in thrombosis. These anions include cell surface mucopolysaccharides, platelet polyphosphates, DNA from endothelial cells, or von Willebrand factor (VWF). Among them, PF4–VWF, as a new immune complex, may induce and promote the formation of immune-associated thrombosis and is expected to become a new target and therapeutic direction. For both HIT and VITT, there is no effective and targeted treatment except discontinuation of suspected drugs. The research and development of targeted drugs based on the mechanism of action have become an unmet clinical need. Here, this study systematically reviewed the characteristics and pathophysiological mechanisms of PF4 and VWF, elaborated the potential mechanism of action of PF4–VWF complex in immune-associated thrombosis, summarized the current status of new drug research and development for PF4 and VWF, and discussed the possibility of this complex as a potential biomarker for early immune-associated thrombosis events. Moreover, the key points of basic research and clinical evaluation are put forward in the study.

## Introduction

After vaccination with recombinant adenovirus vector (ChAdOx1-nCov-19, AstraZeneca) encoding spike protein antigen of severe acute respiratory syndrome coronavirus 2 (SARS CoV-2), several cases of abnormal thrombotic events and thrombocytopenia occurred, known as vaccine-induced immune thrombotic thrombocytopenia (VITT) (1). This suggests a disorder that clinically resembles severe heparin-induced thrombocytopenia (HIT), a well-known prothrombotic disorder caused by platelet-activating antibodies that recognize multimolecular complexes between cationic platelet factor 4 (PF4) and anionic heparin (2). The VITT-affected patients were strongly positive in the PF4/polyanion immunoassay (enzyme-immunoassay (EIA)), and the platelet activation induced by serum was the largest in the presence of PF4 (3).

However, unlike the usual case of HIT, these vaccinated patients did not receive any heparin to explain the subsequent development of thrombosis and thrombocytopenia. How PF4 plays a role in immune-associated coagulation abnormalities has not been clearly determined. More data are needed on the pathogenesis of this abnormal coagulation disorder.

## Source, structure, and characteristics of PF4

**Figure 1:**
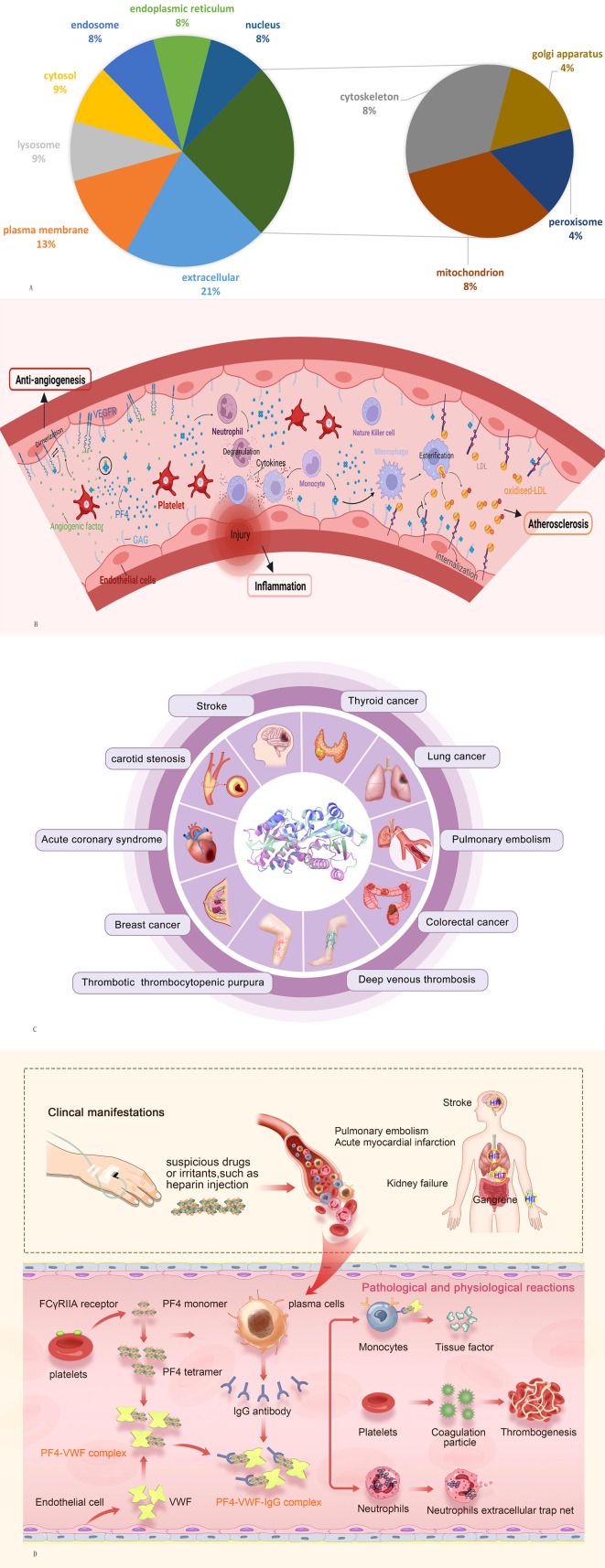
**(A)** Subcellular locations of PF4 gene from compartments. **(B)** Potential function of PF4 in antiangiogenesis, inflammation, and atherosclerosis. **(C)** Overview of systemic diseases involving VWF. **(D)** Potential mechanistic hypothesis of the PF4–VWF complex in inducing and accelerating thrombosis. PF4, platelet factor 4; VWF, von Willebrand factor.

PF4, also known as chemokine (C-X-C motif) ligand 4 (CXCL4), is synthesized by megakaryocytes, internalized into vesicles, and then packaged in platelet α-granules (4, 5). In addition to megakaryocytes, PF4 is also expressed by cultured microglia (6). Moreover, immunoelectron microscopy studies confirmed that PF4 localizes not only to α-granules but also to mast cells (7). When platelets are stimulated by aggregating agents, such as thrombin or adenosine 50-diphosphate (ADP) (8) and arachidonic acid (9), PF4 is released from α-granules. In addition, PF4 is also released by monocytes, neutrophils, and activated T cells (10, 11). Subcellular locations of PF4 gene are summarized in **Figure 1A** (data from GeneCards.org).

PF4 is secreted as a tetramer, that is, comprised of four identical subunits that assemble to form a globular protein (12). The genes encoding human PF4 are located in q13.1 in the global run-on (GRO) region of chromosome 4, which includes a 3′-untranslated region, the entire amino acid coding region for the mature PF4 protein, and a 5′ region containing coding information for an additional 18 amino acids (13, 14). Full-length human PF4 is composed of 101 amino acids, which includes a hydrophobic signal-like sequence involved in transmembrane transport (14) and a mature monomeric human PF4 peptide with a molecular weight of 7.8 kDa containing 70 amino acids (15). Using nuclear magnetic resonance (NMR) spectroscopy, researchers have shown that pH and ionic strength play key roles in the state of PF4. When the pH is approximately 4, lowering the pH shifts the equilibrium to the monomer state, while increasing the pH leads the equilibrium to the dimer and tetramer states. Additionally, increasing the solvent ionic strength stabilizes the tetramer state, particularly at low pH levels (16).

## Potential biological function of PF4

PF4 is released when platelets are activated, and it has been found to play an important role in coagulation, tumors, antithrombotic neovascularization, fibrosis, infectious diseases, and other diseases. Among them, the most important and widely recognized function of PF4 is its participation in the formation of immune-related thrombosis.

Unlike most chemokines that bind to clear receptors, the involvement of specific CXCL receptors in mediating different PF4 activities remains uncertain (17). However, PF4 has a high affinity for polyanions, such as surface mucopolysaccharides, platelet polyphosphates, and DNA from endothelial cells (18). Polyanions (heparin, bacteria, vaccine, etc.) bind to PF4 to form a complex that promotes the release of various cytokines (growth factors, inflammatory factors, and chemokines) and induces immune cells to produce antibodies against the complex (**Table 1**). Then, antibodies can bind to the FcγRIIA receptor on the platelet surface, promoting platelet aggregation and the release of inflammatory factors, ultimately leading to thrombosis (23). However, the formation of this complex is affected by the charge and the concentration of PF4 and polyanions, and when the concentration of PF4 or polyanions increases, the combination could become weakened, and the thrombus-promoting effect is diminished (24). In addition, the PF4 tetramer binds in a calcium-independent manner to the chondroitin sulfate (CS) side-chain on the cell-surface glycosaminoglycan (GAG) domain of thrombomodulin (TM), augmenting activated protein C (APC) formation by up to 25-fold (25). Meanwhile, PF4 is bound to the anionic γ-carboxyglutamic acid (Gla) domain of protein C (PC) (17), ultimately enhancing APC generation by the thrombin–thrombomodulin complex (26). Furthermore, PF4 can neutralize the natural anticoagulant effect of negatively charged GAGs, including heparin (27). PF4 prevents heparin binding to antithrombin III leading to inhibition of heparin-dependent thrombin inactivation (28) and facilitates platelet aggregation even in the presence of suboptimal inducer concentrations (29).

**Table 1:** Potential mechanism of PF4 in different thrombosis diseases.

Category	HIT or classic HIT (19)	Autoimmune HIT (20)	VITT (21, 22)
**Polyanion binding to PF4 (trigger)**	Heparin (UFH/LMWH)	Heparin, bacteria, or pathogen	Adenovirus vector vaccine and the component
**Target cells**	Platelet, neutrophil, monocytes, and B cells	Platelet, platelet microparticles, endothelial cells, and monocytes	Platelet, monocytes, neutrophils, and endothelium cell
**Clinical presentation**	Thrombocytopenia with or without thrombosis; high-titer anti-PF4 antibody	Delayed-onset HIT; persisting HIT; spontaneous HIT; heparin “flush” HIT; fondaparinux-associated HIT; severe HIT (platelet count lower than 20 × 10^9^/L) with associated DIC	High-titer anti-PF4; thrombosis in unusual sites; thrombocytopenia
**Treatments**	Stop heparin; anticoagulation (argatroban/bivalirudin/fondaparinux/DOACs/warfarin/); IVIg or TPE	Heparin cessation and avoidance/reversal of vitamin K antagonists; alternative anticoagulation (subcutaneous fondaparinux/NOAC); high-dose IVIg or TPE	Anticoagulation; modulation of the autoimmune phenomenon; supportive care; organ-specific (surgical) interventions

VITT, vaccine-induced immune thrombotic thrombocytopenia; UFH, unfractionated heparin; LMWH, low-molecular-weight heparin; DOAC, direct oral anticoagulant; IVIg, intravenous immunoglobulin; TPE, therapeutic plasma exchange; DIC, disseminated intravascular coagulation; HIT, heparin-induced thrombocytopenia; NOAC, novel oral anticoagulant.

In addition, PF4 plays an important role in antiangiogenesis, atherosclerosis, the inflammatory response, and tumor biology mainly through its ability to regulate angiogenesis and the function of different immune cell types (**Figure 1B**). PF4 exerts potent angiostatin effects by inhibiting endothelial cell proliferation, and this effect is localized to amino acid residues 17–70 of the molecule (30). PF4 and p17-70 can inhibit proangiogenic factors such as fibroblast growth factor 2 (FGF2) to suppress angiogenesis (31, 32). Moreover, PF4 also inhibits the function of vascular endothelial growth factor (VEGF) by disrupting the binding of VEGF to its receptor and suppressing the VEGF-induced intracellular signaling cascade (33). PF4 stimulates immune cancer surveillance and tumor inhibition by enhancing the adhesion of neutrophils, eosinophils, and monocytes and inhibiting the activation and proliferation of T cells, which decreases metastasis formation and tumor-platelet aggregates in animal models (34, 35). PF4 inhibits low-density lipoprotein (LDL) catabolism by competing for binding to LDL receptors (LDL-Rs) primarily through interaction with cell-associated chondroitin sulfate proteoglycans and disrupting normal endocytic transport of LDL/LDL-R complexes (36). The resulting retention of LDL on the cell surface may promote the formation of oxidized LDL, ultimately supporting an expanded role for platelets in the mechanism of atherosclerotic disease (37). Regarding the inflammatory response, PF4 triggers chemotaxis of human polymorphonuclear leukocytes and monocytes, attracting inflammatory cells to sites of blood vessel injury, promoting neutrophil degranulation, and stimulating cytokine production in monocytes, which act on natural killer cells, neutrophils, mononuclear phagocytes, and T regulatory cells (38, 39). Moreover, PF4 is also involved in the physiopathological process of chronic obstructive pulmonary disease (COPD) (40), pancreatic cancer (41), periodontitis (42), polycystic ovary syndrome (PCOS) (43), and thyroiditis (44).

## Potential role of the PF4–VWF complex in immune-associated thrombosis

Von Willebrand factor (VWF) is a multimeric adhesive protein that is primarily synthesized and stored in endothelial cells, megakaryocytes, and platelet precursors in the bone marrow (45). It is encoded by the VWF gene located on the short arm of chromosome 12 and is involved in the occurrence and development of a variety of systemic abnormal diseases, especially in hemorrhagic and thrombotic conditions (46) (**Figure 1C**). An elevated plasma level of VWF may predict a thrombotic occurrence, while a decreased plasma level may indicate a bleeding condition (47). When a vessel injury occurs, VWF acts as a molecular bridge by promoting platelet adhesion to the subendothelial at sites of vascular injury and platelet–platelet interactions in high shear-rate conditions, contributing to primary hemostasis (48). It is also the carrier of factor VIII (FVIII), indirectly contributing to the coagulation process (49).

The injured vascular endothelial cells release a large number of VWF molecules, which spontaneously assemble into VWF strings under the action of low shear force, providing binding sites for PF4. In 2020, PF4–VWF complexes were first found in patients with thrombotic thrombocytopenic purpura (TTP) (50). As demonstrated, PF4 was incubated with either bovine serum albumin (BSA), GPIIb/IIIa, or VWF, and PF4 interacted with VWF only, indicating that the interaction was specific to VWF. PF4 suppresses ADAMTS13 (a disintegrin and metalloproteinase with a thrombospondin type 1 motif, member 13) activity when it binds to the VWF-A2 domain in a concentration-dependent manner (50), potentially worsening thrombosis. When the ratio was 1:1 (hPF4:VWF = 10:10 µg/ml), the mean mass of the hPF4–VWF complexes widened with an appearance similar to that of VWF alone (51). Moreover, PF4 complexes that bind to VWF chains can be recognized by HIT antibodies, and platelets then bind extensively to these complexes, which is inhibited by monoclonal antibodies that block FcγRIIA or glycoprotein Ib-IX (51). These results indicate that the formation of immune complexes mediated by VWF promotes thrombosis and participates in the occurrence and development of HIT.

Von Willebrand disease (VWD) is a bleeding disorder caused by quantitative (type 1 or 3) or qualitative (type 2A/2B/2M/2N) defects of circulating VWF (52). However, circulating VWF levels do not always fully explain bleeding phenotypes, suggesting a role for alternative factors, like platelets. A positive correlation between PF4 and VWF : Ag levels in type 2A VWD patients (r = 0.229, p = 0.010) was found, which might indicate a similar interaction *in vivo* (53). Low plasma ADAMTS13 activity or reduced ratio of ADAMTS13 activity to VWF antigen or activity was found prevalent in patients with suspected HIT (54), which suggests that PF4 may reduce VWF cleavage by inhibiting ADAMTS13 activity. Similarly, ADAMTS13 levels were reduced (p = 0.009) and the VWF/ADAMTS13 ratio was increased (p = 0.0004) in convalescent COVID-19 patients with dysregulated angiogenesis and immunothrombosis, while levels of PF4 (a putative protector of VWF) were also elevated (p = 0.0001) (55). Moreover, recent findings revealed mortality to be significantly correlated with VWF antigen (r = 0.38; p = 0.0022) and soluble thrombomodulin (r = 0.38; p = 0.0078) among patients with COVID-19-associated coagulopathy (56). As PF4 was found to bind to VWF and protect against ADAMTS13 activity (50), studies suggested that ADAMTS13 activity against VWF was prevented by stabilization/protection of the PF4–VWF complex mediated by PF4 antibodies (57). At present, studies on the interaction between PF4 and VWF are limited, and more studies are needed to focus on the inhibitory effect of PF4 on the activity of ADAMTS13 and the role of the PF4–VWF complex in immune-related thrombotic diseases.

No drugs targeting PF4 have been successfully marketed, and only one HIT diagnostic kit targeting PF4/heparin antibody is available for *in vitro* diagnosis. Drugs that prevent VWF-associated thrombosis include the metalloproteinases ADAMTS13 (58) and *N*-acetylcysteine (59), by digesting high-molecular-weight VWF strings and by preventing their formation, respectively. Thus, therapeutic strategies targeting PF4 and VWF-associated thrombosis are novel methods for the diagnosis and treatment of immune-associated thrombosis. We searched the clinical trials registered on the *Clinical Trial website* and found 48 drugs or devices focusing on the diagnosis and treatment of HIT (PF4-related disease), VWD (VWF-related disease), and other thrombosis diseases (**Supplementary Table 1**). A total of four clinical trials focused on PF4, two of which were medical diagnostic devices and two of which were drugs, in patients with HIT, thrombosis, coronary artery bypass graft surgery presence of heparin/PF4 antibody, and end-stage renal disease. Of the remaining 44 studies, four studies focused on VWF-related diagnostic methods, and 40 reported the treatment of hematological disease. These therapeutic drugs mainly include plasma-derived FVIII/VWF concentrate, VWF replacement therapy with Wilate, tranexamic acid, and biological agents (Advate, Alphanate, BIVV001, Biostate, caplacizumab, recombinant VWF/FVIII, etc.). Thus, there is still a lack of effective direct-blocking drugs for thrombotic events caused by HIT and HIT-like diseases. The discovery of new therapeutic targets has become a clinical problem for disease interpretation, diagnosis, and treatment.

## Conclusions and perspectives

PF4 is a cationic protein that easily forms polymers with polyanions to expose new antigenic sites and stimulate immune-mediated thrombocytopenia and thrombosis. However, in the COVID-19 vaccinated population, severe COVID-19 patients, infection, and some spontaneous HIT case reports, the combination of PF4 with other polyanions to form an antigen complex can induce the production of anti-PF4 antibodies, leading to thrombotic thrombocytopenia, even in the absence of a history of heparin exposure.

Among them, PF4 binds along the surface of elongated strings of VWF polymers, where specific binding sites are exposed by ADAMTS13 cleavage. Thus, we speculate that similar to the mechanism of HIT, VWF, as a polyanion, forms a super-large immune complex with PF4 and induces PF4 to expose neoantigens. The PF4–VWF complex might underlie platelet activation and inflammation that make and exacerbate immune-associated thrombosis (**Figure 1D**): PF4 is released by activated platelets and forms a tetramer after the patient is exposed to suspicious drugs or irritants. At the same time, IgG antibody is released from plasma cells and forms PF4–VWF antigen complexes with the activated endothelial extruded cell surface VWF. PF4–VWF complexes bind with IgG antibody as a supercomplex and then bind with the FcγRIIA receptor, leading to 1) activation, aggregation, and additional release of PF4 and procoagulant particles; 2) tissue factor release by endothelial cells; and 3) formation of neutrophil extracellular traps (NETs) after stimulation of neutrophils. Tissue factors, NETs, and procoagulant particles act on thrombin, leading to platelet aggregation and thrombosis. In future drug and product development, studies focusing on the induction of PF4 conformational changes (PF4V1), blocking the binding of PF4 to VWF, the competitive binding of the complex to FcγRIIA receptors, and the prevention of NET release may effectively prevent thrombosis caused by PF4–VWF complex. In-depth mechanistic and clinical research on the PF4–VWF complex will help broaden the early theory of immune-associated thrombosis pathogenesis and identify new biomarkers for the development of vaccines, new heparin drugs, and *in vitro* diagnostic kits.

In summary, we systematically summarized the possible mechanisms of PF4 and VWF in immune-related thrombosis and proposed potential directions for future basic and clinical translational research.

## Author contributions

All authors listed substantially and directly contributed to the review. All authors contributed to the article and approved the submitted version.
