# Supplementary material for: New perspectives on the induction and acceleration of immune-associated thrombosis by PF4 and VWF
Source: Front Immunol. 2023 Feb 28;14:1098665. doi: 10.3389/fimmu.2023.1098665 (PMC10011124; doi:10.3389/fimmu.2023.1098665)
Supplement: Supplementary file 1 [file Table_1.docx]

Supplementary Table 1 Clinical registration studies of new drugs or devices related to PF4 and VWF.

|  | NCT Number | Conditions | Interventions | Characteristics | Population | Sponsor/ Collaborators | Dates of Study Start and Completion |
| --- | --- | --- | --- | --- | --- | --- | --- |
| 1 | NCT03148912 | Heparin-induced Thrombocytopenia (HIT) | Diagnostic Test: Diagnostic algorithm | Study Type: Interventional Phase: Not Applicable Study Design: • Allocation: Not Applicable • Intervention Model: Single Group Assignment • Masking: None (Open Label) • Primary Purpose: Diagnostic | Enrollment: 180 Age: 18 Years and older (Adult, Older Adult) Sex: All | Ottawa Hospital Research Institute | November 23, 2018-December 2022 |
| 2 | NCT00237328 | Thrombosis | Drug: Heparin | Study Type: Observational Phase: Not Applicable Study Design: • Observational Model: Cohort • Time Perspective: Prospective | Enrollment: 1015 Age: 18 Years and older (Adult, Older Adult) Sex: All | Duke University | June 2006-July 2012 |
| 3 | NCT01246011 | Coronary Artery Bypass Graft Surgery Presence of Heparin/Platelet Factor 4 Antibody | Drug: Argatroban and warfarin | Study Type: Interventional Phase: Phase 4 Study Design: • Allocation: Randomized • Intervention Model: Factorial Assignment • Masking: None (Open Label) • Primary Purpose: Other | Enrollment: 9 Age: 18 Years and older (Adult, Older Adult) Sex: All | Massachusetts General Hospital GlaxoSmithKline | Study Start: November 2010 Study Completion: July 2011 |
| 4 | NCT02165761 | End Stage Renal Disease | Device: GORE® Hybrid Vascular Graft Device: Non- heparin bonded synthetic graft | Study Type: Interventional Phase: Not Applicable Study Design: • Allocation: Randomized • Intervention Model: Parallel Assignment • Masking: None (Open Label) • Primary Purpose: Basic Science | Enrollment: 42 Age: 18 Years and older (Adult, Older Adult) Sex: All | W.L.Gore & Associates | Study Start: July 2014 Study Completion: January 20, 2017 |
| 1 | NCT05116501 | Low Von Willebrand Factor | Diagnostic Test: Whole-exome sequencing | Study Type: Interventional Phase: Not Applicable Study Design: • Allocation: Non- Randomized • Intervention Model: Single Group Assignment • Masking: None (Open Label) • Primary Purpose: Basic Science | Enrollment:300 Age: 18 Years to 80 Years (Adult, Older Adult) Sex: All | Fondazione IRCCS Ca' Granda, Ospedale Maggiore Policlinico | Study Start: March 1, 2022 Study Completion: May 30, 2023 |
| 2 | NCT03728049 | Aortic Valve Stenosis Aortic Valve Insufficiency | Diagnostic Test: CT-ADP performed during TAVI procedure Other: No CT-ADP performed during TAVI procedure | Study Type: Interventional Phase: Not Applicable Study Design: • Allocation: Randomized • Intervention Model: Parallel Assignment • Masking: None (Open Label) • Primary Purpose: Treatment | Enrollment: 944 Age: 18 Years and older (Adult, Older Adult) Sex: All | University Hospital, Lille Siemens Healthineers, France Ministry of Health, France | Study Start: December 18, 2019 Study Completion: December 2022 |
| 3 | NCT04317469 | ARDS, Human | Diagnostic Test: VWF measurement | Study Type: Observational Phase: Not Applicable Study Design: • Observational Model: Case-Only • Time Perspective: Prospective | Enrollment: 60 Age: 18 Years to 80 Years (Adult, Older Adult) Sex: All | Ain Shams University | Study Start: March 21, 2020 Study Completion: September 20, 2021 |
| 4 | NCT05008458 | Febrile Seizure | Diagnostic Test: von willebrand factor and copeptin | Study Type: Observational Phase: Not Applicable Study Design: • Observational Model: Case-Control • Time Perspective: Cross- Sectional | Enrollment: 90 Age: 6 Months to 6 Years (Child) Sex: All | Sohag University | Study Start: September 1, 2021 Study Completion: May 15, 2022 |
| 5 | NCT03204539 | Hemophilia A with Inhibitor Hemophilia A | Drug: Wilate | Study Type: Interventional Phase: Phase 4 Study Design: • Allocation: Randomized • Intervention Model: Parallel Assignment • Masking: Quadruple (Participant, Care Provider, Investigator, Outcomes Assessor) • Primary Purpose: Treatment | Enrollment: 1 Age: Child, Adult, Older Adult Sex: Male | University of California, Davis | Study Start: June 1, 2017 Study Completion: July 19, 2019 |
| 6 | NCT00555555 | Von Willebrand Disease | Biological: Alphanate SD/HT | Study Type: Interventional Phase: Phase 4 Study Design: • Allocation: Not Applicable • Intervention Model: Single Group Assignment • Masking: None (Open Label) • Primary Purpose: Treatment | Enrollment: 15 Age: 7 Years and older (Child, Adult, Older Adult) Sex: All | Grifols Biologicals, LLC | Study Start: September 2007 Study Completion: March 2029 |
| 7 | NCT02479087 | Hemophilia A | Drug: Plasma- derived FVIII/VWF concentrate | Study Type: Interventional Phase: Phase 4 Study Design: • Allocation: Not Applicable • Intervention Model: Single Group Assignment • Masking: None (Open Label) • Primary Purpose: Treatment | Enrollment: 20 Age: up to 12 Years (Child) Sex: Male | Fondazione IRCCS Ca' Granda, Ospedale Maggiore Policlinico Sintesi Research Srl | Study Start: January 2015 Study Completion: January 2020 |
| 8 | NCT03205163 | Hemophilia A | Biological: Advate (Low Dose, High Dose) BIVV001 (Low Dose, High Dose) | Study Type: Interventional Phase: Phase 1, Phase 2 Study Design: • Allocation: Non- Randomized • Intervention Model: Sequential Assignment • Masking: None (Open Label) • Primary Purpose: Other | Enrollment: 16 Age: 18 Years to 65 Years (Adult, Older Adult) Sex: Male | Bioverativ, a Sanofi company Sanofi | Study Start: August 28, 2017 Study Completion: November 12, 2018 |
| 9 | NCT04146376 | Von Willebrand Diseases | Other: Use of a postpartum diary and additional blood draws Drug: VWF replacement therapy with Wilate Drug: Tranexamic acid Other: Use of a postpartum diary and additional blood draws. | Study Type: Observational Phase: Not Applicable Study Design: • Observational Model: Cohort • Time Perspective: Prospective | Enrollment: 110 Age: 18 Years and older (Adult, Older Adult) Sex: Female | Bloodworks Mary M. Gooley Hemophilia Center Ergomed bOctapharma | Study Start: October 12, 2019 Study Completion: June 2023 |
| 10 | NCT01151423 | Acquired Thrombotic Thrombocytopenic Purpura | Biological: Caplacizumab Biological: Placebo | Study Type: Interventional Phase: Phase 2 Study Design: • Allocation: Randomized • Intervention Model: Parallel Assignment • Masking: Single (Participant) • Primary Purpose: Treatment | Enrollment: 75 Age: 18 Years and older (Adult, Older Adult) Sex: All | Ablynx | Study Start: January 2011 Study Completion: March 2014 |
| 11 | NCT04161495 | Factor VIII Deficiency | Drug: efanesoctocog alfa (BIVV001) | Study Type: Interventional Phase: Phase 3 Study Design: • Allocation: Non- Randomized • Intervention Model: Parallel Assignment • Masking: None (Open Label) • Primary Purpose: Treatment | Enrollment: 159 Age: 12 Years and older (Child, Adult, Older Adult) Sex: All | Bioverativ, a Sanofi company Sanofi | Study Start: November 19, 2019 Study Completion: February 3, 2022 |
| 12 | NCT01051544 | Severe Hemophilia A | Drug: FVIII Concentrates Drug: FVIII/VWF concentrates | Study Type: Interventional Phase: Not Applicable Study Design: • Allocation: Randomized • Intervention Model: Parallel Assignment • Masking: None (Open Label) • Primary Purpose: Prevention | Enrollment: 0 Age: Child, Adult, Older Adult Sex: Male | City of Hope Medical Center Charta Foundation Grifols Biologicals, LLC CSL Behring Biotest Pharmaceuticals Corporation Grifols Therapeutics LLC | Study Start: September 25, 2009 Study Completion: June 25, 2020 |
| 13 | NCT00168090 | Von Willebrand Disease Blood Coagulation Disorders Blood Platelet Disorders Hematologic Disease | Drug: Blood coagulation Factor VIII and vWF, human | Study Type: Interventional Phase: Phase 4 Study Design: • Allocation: Non- Randomized • Intervention Model: Single Group Assignment • Masking: None (Open Label) • Primary Purpose: Treatment | Enrollment: 30 Age: Child, Adult, Older Adult Sex: All | CSL Behring | Study Start: October 2001 Study Completion: May 2006 |
| 14 | NCT03613584 | Acquired Von Willebrand Disease | Drug: Von Willebrand Factor Drug: Saline Solution | Study Type: Interventional Phase: Phase 2 Study Design: • Allocation: Randomized • Intervention Model: Parallel Assignment • Masking: Double (Participant, Investigator) • Primary Purpose: Treatment | Enrollment: 68 Age: 18 Years and older (Adult, Older Adult) Sex: All | Tirol Kiniken GmbH LFB BIOMEDICAME | Study Start: April 16, 2018 Study Completion: March 10, 2021 |
| 15 | NCT02628509 | Heart Failure Aortic Stenosis | Device: cardiac devices | Study Type: Observational Phase: Not Applicable Study Design: • Observational Model: Cohort • Time Perspective: Prospective | Enrollment: 500 Age: 18 Years and older (Adult, Older Adult) Sex: All | University Hospital, Lille | Study Start: August 2012 Study Completion: January 2018 |
| 16 | NCT04555785 | Bleeding | Drug: Wilate Other: Placebo | Study Type: Interventional Phase: Phase 4 Study Design: • Allocation: Randomized • Intervention Model: Parallel Assignment • Masking: Triple (Participant, Care Provider, Outcomes Assessor) • Primary Purpose: Treatment | Enrollment: 120 Age: 18 Years and older (Adult, Older Adult) Sex: All | University Hospital, Basel, Switzerland | Study Start: April 1, 2022 Study Completion: March 2025 |
| 17 | NCT02283268 | Von Willebrand Disease | Biological: Recombinant von Willebrand Factor (rVWF) | Study Type: Interventional Phase: Phase 3 Study Design: • Allocation: Not Applicable • Intervention Model: Single Group Assignment • Masking: None (Open Label) • Primary Purpose: Treatment | Enrollment: 24 Age: 18 Years and older (Adult, Older Adult) Sex: All | Baxalta now part of Shire Takeda | Study Start: April 1, 2015 Study Completion: July 6, 2016 |
| 18 | NCT00941616 | Von Willebrand Disease | Biological: Biostate® | Study Type: Interventional Phase: Phase 2, Phase 3 Study Design: • Allocation: Non- Randomized • Intervention Model: Parallel Assignment • Masking: None (Open Label) • Primary Purpose: Treatment | Enrollment: 22 Age: 12 Years and older (Child, Adult, Older Adult) Sex: All | CSL Behring Parexel | Study Start: June 2009 Study Completion: February 2012 |
| 19 | NCT00816660 | Von Willebrand Disease | Biological: Recombinant von Willebrand factor : recombinant FVIII (rVWF:rFVIII) Biological: Marketed plasma- derived VWF/FVIII concentrate | Study Type: Interventional Phase: Phase 1 Study Design: • Allocation: Randomized • Intervention Model: Crossover Assignment • Masking: Single (Participant) • Primary Purpose: Other | Enrollment: 32 Age: 18 Years to 60 Years (Adult) Sex: All | Baxalta now part of Shire Takeda | Study Start: December 1, 2008 Study Completion: August 31, 2010 |
| 20 | NCT00632242 | Purpura, Thrombotic Thrombocytopenic Von Willebrand Disease Type-2b | Drug: ARC1779 | Study Type: Interventional Phase: Phase 2 Study Design: • Allocation: Non- Randomized • Intervention Model: Single Group Assignment • Masking: None (Open Label) • Primary Purpose: Treatment | Enrollment: 28 Age: 18 Years to 75 Years (Adult, Older Adult) Sex: All | Archemix Corp. | Study Start: January 2008 Study Completion: December 2008 |
| 21 | NCT01064284 | Hemophilia A | Drug: PLASMA DERIVED Factor VIII Drug: Recombinant FVIII | Study Type: Interventional Phase: Phase 4 Study Design: • Allocation: Randomized • Intervention Model: Parallel Assignment • Masking: None (Open Label) • Primary Purpose: Basic Science | Enrollment: 303 Age: 1 Minute to 6 Years (Child) Sex: Male | Fondazione Angelo Bianchi Bonomi Sintesi Research Srl | Study Start: January 2010 Study Completion: May 2015 |
| 22 | NCT01410227 | Von Willebrand Disease | Biological: Recombinant von Willebrand factor (rVWF) Drug: Placebo Biological: Recombinant factor VIIII (rFVIII) | Study Type: Interventional Phase: Phase 3 Study Design: • Allocation: Non- Randomized • Intervention Model: Crossover Assignment • Masking: None (Open Label) • Primary Purpose: Treatment | Enrollment: 49 Age: 18 Years to 65 Years (Adult, Older Adult) Sex: All | Baxalta now part of Shire Takeda | Study Start: November 1, 2011 Study Completion: February 1, 2014 |
| 23 | NCT03095287 | Hemophilia A, Congenital | Biological: Alphanate | Study Type: Interventional Phase: Phase 2 Study Design: • Allocation: Not Applicable • Intervention Model: Single Group Assignment • Masking: None (Open Label) • Primary Purpose: Treatment | Enrollment: 2 Age: up to 12 Years (Child) Sex: Male | Grifols Therapeutics LLC Grifols Biologicals, LLC | Study Start: January 3, 2018 Study Completion: September 18, 2020 |
| 24 | NCT04106908 | VWD - Von Willebrand's Disease | Biological: Eqwilate | Study Type: Observational Phase:  Not Applicable     Study Design: • Observational Model: Case-Only • Time Perspective: Prospective | Enrollment: 47 Age: 6 Years and older (Child, Adult, Older Adult) Sex: All | Octapharma | Study Start: November 27, 2019 Study Completion: September 2021 |
| 25 | NCT01365546 | Prevent Bleeding in Major Surgery | Biological: human VWF/FVIII concentrate | Study Type: Interventional Phase: Phase 3 Study Design: • Allocation: Not Applicable • Intervention Model: Single Group Assignment • Masking: None (Open Label) • Primary Purpose: Prevention | Enrollment: 30 Age: 6 Years and older (Child, Adult, Older Adult) Sex: All | Octapharma | Study Start: June 2011 Study Completion: April 2014 |
| 26 | NCT00879541 | Hemophilia A | Biological: Biostate® [SP] Biological: Biostate® [RP] | Study Type: Interventional Phase: Phase 2 Study Design: • Allocation: Randomized • Intervention Model: Crossover Assignment • Masking: Double (Participant, Investigator) • Primary Purpose: Treatment | Enrollment: 81 Age: 12 Years and older (Child, Adult, Older Adult) Sex: Male | CSL Behring Parexel | Study Start: February 2009 Study Completion: October 2010 |
| 27 | NCT04770935 | Von Willebrand's Disease (VWD) | Drug: efanesoctocog alfa (BIVV001) | Study Type: Interventional Phase: Phase 1 Study Design: • Allocation: Not Applicable • Intervention Model: Single Group Assignment • Masking: None (Open Label) • Primary Purpose: Other | Enrollment: 9 Age: 18 Years to 65 Years (Adult, Older Adult) Sex: All | Bioverativ, a Sanofi company Sanofi | Study Start: May 3, 2021 Study Completion: May 25, 2023 |
| 28 | NCT04644575 | Hemophilia A | Drug: efanesoctocog alfa (BIVV001) | Study Type: Interventional   Phase: Phase 3 Study Design: • Allocation: Non- Randomized • Intervention Model: Single Group Assignment • Masking: None (Open Label) • Primary Purpose: Treatment | Enrollment: 262 Age: Child, Adult, Older Adult Sex: All | Bioverativ, a Sanofi company Sanofi | Study Start: February 23, 2021                          Study Completion: February 12, 2027 |
| 29 | NCT04759131 | Hemophilia A | Drug: efanesoctocog alfa (BIVV001) | Study Type: Interventional Phase: Phase 3 Study Design: • Allocation: Not Applicable • Intervention Model: Single Group Assignment • Masking: None (Open Label) • Primary Purpose: Treatment | Enrollment: 75 Age: up to 12 Years (Child) Sex: Male | Bioverativ, a Sanofi company Sanofi | Study Start: February 19, 2021 Study Completion: February 1, 2023 |
| 30 | NCT01051076 | Severe Hemophilia A | Drug: VWF/FVIII concentrates | Study Type: Interventional Phase: Not Applicable Study Design: • Allocation: Non- Randomized • Intervention Model: Single Group Assignment • Masking: None (Open Label) • Primary Purpose: Treatment | Enrollment: 3 Age: Child, Adult, Older Adult Sex: Male | City of Hope Medical Center Charta Foundation Grifols Biologicals, LLC CSL Behring Biotest Pharmaceuticals Corporation Grifols Therapeutics LLC | Study Start: November 3, 2009 Study Completion: October 21, 2020 |
| 31 | NCT00387192 | Von Willebrand Disease | Drug: Optivate | Study Type: Interventional Phase: Phase 3 Study Design: • Allocation: Non- Randomized • Intervention Model: Single Group Assignment • Masking: None (Open Label) • Primary Purpose: Treatment | Enrollment: 26 Age: 12 Years and older (Child, Adult, Older Adult) Sex: All | Bio Products Laboratory | Study Start: November 2006 Study Completion: September 2008 |
| 32 | NCT03879135 | Von Willebrand Disease (VWD) | Biological: rVWF Biological: rFVIII | Study Type: Interventional Phase: Phase 3 Study Design: • Allocation: Non- Randomized • Intervention Model: Parallel Assignment • Masking: None (Open Label) • Primary Purpose: Prevention | Enrollment: 71 Age: Child, Adult, Older Adult Sex: All | Baxalta now part of Shire Takeda Development Center Americas, Inc. Takeda | Study Start: April 1, 2019 Study Completion: December 31, 2025 |
| 33 | NCT02932618 | Von Willebrand Disease | Biological: von Willebrand factor (Recombinant) Biological: Antihemophilic Factor (Recombinant) | Study Type: Interventional Phase: Phase 3 Study Design: • Allocation: Non- Randomized • Intervention Model: Parallel Assignment • Masking: None (Open Label) • Primary Purpose: Treatment | Enrollment: 34 Age: up to 17 Years (Child) Sex: All | Baxalta now part of Shire Takeda Development Center Americas, Inc. Takeda | Study Start: December 18, 2017 Study Completion: March 31, 2023 |
| 34 | NCT04344860 | Von Willebrand Diseases Postpartum Hemorrhage | Drug: Recombinant Von Willebrand factor Drug: Tranexamic Acid Injection [Cyklokapron] | Study Type: Interventional Phase: Phase 3 Study Design: • Allocation: Randomized • Intervention Model: Parallel Assignment • Masking: None (Open Label) • Primary Purpose: Prevention | Enrollment: 20 Age: 18 Years and older (Adult, Older Adult) Sex: Female | Margaret Ragni University of Pittsburgh | Study Start: June 4, 2021 Study Completion: September 2023 |
| 35 | NCT02606045 | Von Willebrand Diseases | Drug: recombinant von Willebrand factor Drug: tranexamic acid | Study Type: Interventional Phase: Phase 3 Study Design: • Allocation: Randomized • Intervention Model: Crossover Assignment • Masking: None (Open Label) • Primary Purpose: Treatment | Enrollment: 60 Age: 13 Years to 45 Years (Child, Adult) Sex: Female | Margaret Ragni University of North Carolina Duke University University of Pittsburgh | Study Start: February 7, 2019 Study Completion: December 31, 2022 |
| 36 | NCT02472665 | Von Willebrand Disease | Drug: plasma- derived FVIII/VWF concentrate | Study Type: Interventional Phase: Phase 4 Study Design: • Allocation: Not Applicable • Intervention Model: Single Group Assignment • Masking: None (Open Label) • Primary Purpose: Treatment | Enrollment:8 Age: 2 Months to 6 Years (Child) Sex: All | Grifols Therapeutics LLC Instituto Grifols, S.A. | Study Start: December 2013 Study Completion: December 2024 |
| 37 | NCT00404300 | Von Willebrand Disease | Drug: Optivate | Study Type: Interventional Phase: Phase 3 Study Design: • Allocation: Non- Randomized • Intervention Model: Single Group Assignment • Masking: None (Open Label) • Primary Purpose: Treatment | Enrollment: 25 Age: 12 Years and older (Child, Adult, Older Adult) Sex: All | Bio Products Laboratory | Study Start: February 2007 Study Completion: September 2008 |
| 38 | NCT01445197 | Hemophilia A | Biological: Biostate | Study Type: Interventional Phase: Phase 3 Study Design: • Allocation: Not Applicable • Intervention Model: Single Group Assignment • Masking: None (Open Label) • Primary Purpose: Treatment | Enrollment: 1 Age: 28 Days to 11 Years (Child) Sex: Male | CSL Behring | Study Start: December 2012 Study Completion: December 2013 |
| 39 | NCT04657887 | Von Willebrand Disease | Biological: Voncento® | Study Type: Observational Phase: Study Design: • Observational Model: Cohort • Time Perspective: Prospective | Enrollment: 135 Age: Child, Adult, Older Adult Sex: All | CSL Behring | Study Start: November 23, 2015 Study Completion: December 2023 |
| 40 | NCT02506023 | Hemophilia A | Drug: Desmopressin | Study Type: Interventional Phase: Phase 1 Study Design: • Allocation: Non- Randomized • Intervention Model: Parallel Assignment • Masking: None (Open Label) • Primary Purpose: Diagnostic | Enrollment: 2 Age: 18 Years to 60 Years (Adult) Sex: Female | Emory University | Study Start: July 2015 Study Completion: June 15, 2018 |
| 41 | NCT00742612 | Intracranial Embolism Cerebral Thromboembolism Carotid Stenosis | Drug: ARC1779 Injection Drug: Placebo (normal saline) | Study Type: Interventional Phase: Phase 2 Study Design: • Allocation: Randomized • Intervention Model: Parallel Assignment • Masking: Triple (Participant, Care Provider, Investigator) • Primary Purpose: Treatment | Enrollment: 100 Age: 18 Years to 80 Years (Adult, Older Adult) Sex: All | Archemix Corp. St George's, University of London | Study Start: February 2009 Study Completion: April 2010 |
| 41 | NCT00726544 | Thrombotic Microangiopathy Thrombotic Thrombocytopenic Purpura | Drug: ARC 1779 Placebo Drug: ARC1779 Injection | Study Type: Interventional Phase: Phase 2 Study Design: • Allocation: Randomized • Intervention Model: Parallel Assignment • Masking: Quadruple (Participant, Care Provider, Investigator, Outcomes Assessor) • Primary Purpose: Treatment | Enrollment: 100 Age: 18 Years to 75 Years (Adult, Older Adult) Sex: All | Archemix Corp. | Study Start: December 2008 Study Completion: March 2011 |
| 42 | NCT00524342 | Von Willebrand Disease | Drug: Oprelvekin, Interleukin 11, IL-11 | Study Type: Interventional Phase: Phase 2 Study Design: • Allocation: Not Applicable • Intervention Model: Single Group Assignment • Masking: None (Open Label) • Primary Purpose: Prevention | Enrollment: 7 Age: 18 Years to 45 Years (Adult) Sex: Female | Margaret Ragni University of North Carolina Wyeth is now a wholly owned subsidiary of Pfizer University of Pittsburgh | Study Start: January 2008 Study Completion: December 2010 |
| 43 | NCT00524225 | Von Willebrand Disease | Drug: Neumega (Oprelvekin, Interleukin 11, IL-11) | Study Type: Interventional Phase: Phase 2 Study Design: • Allocation: Not Applicable • Intervention Model: Single Group Assignment • Masking: None (Open Label) • Primary Purpose: Prevention | Enrollment: 3 Age: 18 Years and older (Adult, Older Adult) Sex: All | Margaret Ragni University of North Carolina Wyeth is now a wholly owned subsidiary of Pfizer University of Pittsburgh | Study Start: February 2008 Study Completion: June 2012 |
| 44 | NCT02460458 | Type 3 Von Willebrand's Disease | Drug: Von Willebrand Factor | Study Type: Observational Phase: Not Applicable Study Design: • Observational Model: Cohort • Time Perspective: Other | Enrollment: 265 Age: Child, Adult, Older Adult Sex: All | Fondazione Angelo Bianchi Bonomi Sintesi Research Srl | Study Start: November 5, 2012 Study Completion: December 2022 |
